# Using Identity-Based Cryptography as a Foundation for an Effective and Secure Cloud Model for E-Health

**DOI:** 10.1155/2022/7016554

**Published:** 2022-04-25

**Authors:** Shikha Mittal, Ankit Bansal, Deepali Gupta, Sapna Juneja, Hamza Turabieh, Mahmoud M Elarabawy, Ashish Sharma, Zelalem Kiros Bitsue

**Affiliations:** ^1^Chitkara University Institute of Engineering and Technology, Chitkara University, Punjab, Rajpura, India; ^2^KIET Group of Institutions, Delhi NCR, Ghaziabad, India; ^3^Department of Information Technology, Taif University, College of Computing and Information Technology, P.O. Box 11099, Taif 21944, Saudi Arabia; ^4^Department of Mathematics, Faculty of Science, Suez Canal University, Ismailia 41522, Egypt; ^5^Department of Computer Science, College of Computing and Information Technology, Taif University, P.O. Box 11099, Taif 21944, Saudi Arabia; ^6^Indian Institute of Information Technology, Kota, India; ^7^USAHO, Oromiya, Ethiopia

## Abstract

Nowadays, one of the most popular applications is cloud computing for storing data and information through World Wide Web. Since cloud computing has become available, users are rapidly increasing. Cloud computing enables users to obtain a better and more effective application at a lower cost in a more satisfactory way. Health services data must therefore be kept as safe and secure as possible because the release of this data could have serious consequences for patients. A framework for security and privacy must be employed to store and manage extremely sensitive data. Patients' confidential health records have been encrypted and saved in the cloud using cypher text so far. To ensure privacy and security in a cloud computing environment is a big issue. The medical system has been designed as a standard, access of records, and effective use by medical practitioners as required. In this paper, we propose a novel algorithm along with implementation details as an effective and secure E-health cloud model using identity-based cryptography. The comparison of the proposed and existing techniques has been carried out in terms of time taken for encryption and decryption, energy, and power. Decryption time has been decreased up to 50% with the proposed method of cryptography. As it will take less time for decryption, less power is consumed for doing the cryptography operations.

## 1. Introduction

Today, the field of health care in India is facing major challenges because the technology growth in this field is minimum, and it is essential to provide a complete history of the patient to the doctor. The doctor required the complete details of the patient for proper diagnosis, but it is difficult to maintain the record of each patient and their previous treatment details. Many people undergo treatment for different diseases from different fields of doctors, so the details of the treatments are scattered among the offices, and it is critical to inform treatment details to individual doctors. There are chances of repeating the tests without proper consulting, which is a waste of time and money. Sometimes various combinations of medicines lead to serious health issues. So, accurate record keeping is necessary in the field of health care. The information transferred among the health care doctors through the papers or personal communication in the current methodology made the chances of producing death. E-health care provides the chance to keep all the medical records and the records can be accessed by the doctors during the patient visit with the permission of the patient. The service is utilized for effective maintenance of the health record. The system is also utilized to provide real time information. Electronic health record (EHR) is also known by the name of Electronic Medical Record (EMR) and the records utilize cloud servers for high quality infrastructure with cost benefit. Electronic storage system reduces the chances of utilizing hard copies of records and the format of softcopy can be shared among the surgeons, insurance companies, and third-party administrators. Maintaining the confidentiality and privacy of the patients are the important security issues of electronic health record (EHR) maintenance [1]. Cloud service providers are maintaining and managing the cloud servers, so the environment is a trusty one. As one of the chief objectives of cloud computing providers, cloud security is among their main priorities. Should data kept in cloud resources be stolen or rendered inaccessible for long time as a result of deteriorating network connection, a significant loss of commercial revenue for the user is occurring. However, the company's presence is put in jeopardy because of the unexpected lack of IT services. Research on cloud computing safety hazards, possible mitigation measures, and the best cloud security systems are critical to developing trust in technology and increasing security. It is necessary to store sensitive information related to the patient's medical history a secure way. An accessible resource is offered on the Internet vigorously. The process of encryption becomes an effective and promising technique for protecting information before outsourcing. Also, the EHR owner selects the encryption technique and allows the users to access a file. They have the right to let or cancel the access, and in turn, an authorized user can access the record for any individual or proficient determinations. This helps to classify the public and private users separately. Here, the family members and friends come under the category of personal users, whereas other persons such as nurses, insurance agents, etc., come under the category of public users. More specifically, the paper focuses on the following research questions:

1. To propose a Role Based Proxy Decryption for energy efficiency in Mobile Cloud Computing
2. To compare energy consumption of proposed algorithm with existing algorithms

## 2. Literature Survey

Researchers in the field of E-health cloud computing have done studies based on previous research papers and literature. Some of them are as follows.

Clients can convey information from far away regions to other distant or fixed areas using versatile information correspondence, which has become a fundamental and fast progressing innovation. This turns out to be the solution to the most pressing issue facing financial professionals today: mobility. In this era of wireless communication, however, mobile devices are limited by their processing power, storage, and, most importantly, battery life [2, 3]. The most often utilized applications on mobile devices or other portable devices are chatting and sending texts. However, because the Internet is an open and vulnerable network, some people are concerned about sending sensitive information over it. Encryption- Decryption is the process of making data secure for transmission. Encryption, on the other hand, is computationally costly and consumes energy and computational resources in wireless devices, both of which are restricted. Wireless transmission and larger key sizes impact the power consumption of mobile devices the most [4–7]. Cloud based medical care registering has changed the substance of medical care in numerous ways [8]. The critical advantages of distributed computing in medical care are the versatility of the administrations and the works to upscale or decrease the information storage and intrigue Artificial Intelligence (AI) and AI. Reference [9] proposed a blockchain-based IoT design to give improved security of medical care information by utilizing Identity-Based Encryption (IBE) calculation. The effective sharing of Electronic Health Record (EHR) can further develop the treatment cycle, finding exactness, security, and protection. In [10], cloud computing security and privacy were discussed in detail by the authors. Using cryptography techniques, they have made cloud computing more secure. But some of the limitations consist in a cloud environment for that they offered the homomorphic encryption techniques are used to perform a high level of security, scalable and efficient security in these solutions. Then it required lengthy computations. Attribute-based data sharing approaches for mobile resource users in clouds have been proposed in [11]. It was also possible to verify the correctness of cypher texts before decrypting them online and offline. The security model with the DBDH assumption was selected by the selection, and the attributes set and security model were demonstrated to be secure, even if adding the public parameters lowered the task's offline computing time. In [12], the authors proposed methods of profile matching and safe data sharing in the MHSN cloud computing. Healthcare had, however, altered social networks and cloud computing to allow real-phase data exchange in a cost-effective manner. There were a variety of security problems associated with the increased use of mobile healthcare social networks (MHSN). As a result, patients had access to IBBE (Identity-based broadcast encryption)-encrypted health records from which they can securely communicate with a team of surgeons. The attribute-based reencryption provided in advance allowed clinicians to meet established conditions in ciphertext to be permitted in the cloud environment to transform the data into new, identity-based encryption structures. The MHSN matching process was based on identity encryption and the test of equality as well, which was provided by them as well. It helped the patients to find the networks in the privacy conserving mode and its flexible approval to encrypt the health records with the keywords of guessing attacks. Then the results protected the data in privacy and security with MHSN. In [13], the authors have developed ontologies for controlling, governing, and protecting cloud applications. They have created classifications of threats that cloud operators may encounter and have included them in security models. Cloud security rules and compliance providers were formulated and found through the use of technology by cloud consumers, as an example. Cloud providers' security measures were being defied by users' high levels of security, which were chosen automatically. As a result, the ontology for describing security threats, controls, providers' data, and cloud security rules was semantically built. They pushed for consumers who wanted to move their data to the cloud and secure their worries in order to make cloud security standards more user-friendly. The ontology rules were designed as a reason for a better match between compliant providers and ontology rules. In order to increase cloud security, [14] claimed that the basic methodology and flexibility were necessary. The customer then permits the classification and representation of security requirements. Cloud secSLAs have so far been used to access and evaluate the security level of these advanced techniques as state-of-the-art security. SecSLAs have specified standardization and state-of-the-art works since their inception. Acquire the cloud security alliance's proven techniques based on real-world data from service providers. They emphasized security in pattern as the primary factor in decision-making, and they put into practice strategies for visibly matching the results of CSP-based secSLAs with consumers. However, the technique used to solve this work is going to be recommended as advanced secSLA's and notations such as end-to-end process, uncertainty, and dependencies in secSLA's are involved for better assessment to increase the security assurance in the future. An identity-based encryption framework had been proposed in [15] for secure correspondence between objects remembered for the Internet of Things (IoT). As IoT has been growing quickly with the utilization of new advances today. As well as offering new applications that make our lives simpler, it likewise uncovers genuine security and protection issues. Many sensors around us or the applications we use can impart our private information to different items without our insight. Encryption frameworks can be utilized to forestall this arising security issue. Notwithstanding, it is hard to decide the fitting encryption framework because of restricted power assets and computational abilities. In [16], the authors proposed an effective personality based appropriated unscrambling plan for the PHR framework that does not need to recreate the decoding key. Also, through proposed conspire, it is advantageous to impart their information to numerous gatherings. In [17], to address the symmetrical aspects of cloud security, they conducted a literature review of currently available approaches. In order for cloud professionals to demonstrate to their clients and measure the security of cloud-based client services, they conducted a security assessment. As a result, they advocated the construction of a cloud security software framework and the use of fuzzy system principles to address many of the cloud's security concerns at a higher framework level. In [18], they used geo-distributed clouds to provide the E-health monitoring system with privacy preservation and minimal service delay. Consequently, the system was able to disperse the cloud servers and it was allocated a set of servers to request that the user load-balance the workload. Delays occurred when there were few people using the service. The traffic shaping algorithm was also mentioned. It has the capacity to assess traffic attacks and mitigate them by converting user health data collisions to nonhealth data collisions. The author presented an original totally secure AMRIBE scheme in [19], which is an exquisite scheme that accomplishes the IND-MID-CCA and ANON-MID-CCA security in the standard model with tight reduction. When compared to other existing systems, multireciever identity-based encryption has a cheap cost of encryption. Many applications, such as pay-per-view television, video conferencing, and remote learning require encryption. Authors in [20] stated that the Improved Identity Based Encryption method provides low power consumption, good encryption stability, and high authentication potential, making it ideal for implementation in wireless sensor networks with high security needs. In [21], creators joined the ideas of key-protected encryption (KIE) and personality based encryption with the uniformity test (IBE-ET) to acquire character-based key-protected encryption with correspondence test (IB-KIEET) that acquired the benefits of personality based encryption (IBE), which improves on the declaration the executives for public key encryption. Besides, the key-protected component was added to lessen the chance of key openness. Presented and formalized in [22], a character-based encryption change (IBET) model via flawlessly incorporating two encryption instruments, to be specific IBE and personality-based transmission encryption (IBBE). In IBET, information clients are distinguished and approved for information access in light of their unmistakable characters, which keeps away from muddled testament the board in normal, secure appropriated frameworks.

In [23, 24], the authors had discussed and defined about Role based encryption, Role based proxy reencryption, and Role based proxy decryption level-I. Result analysis and performance also had been done for the same on the basis of tome and cost. Now, further work has been extended to define the Role based proxy decryption level-II. In this work, performance analysis of consumed energy in terms of time also has been analyzed for existing methods and proposed methods.

## 3. Materials and Methods

Cryptography is based on complex mathematical operations like prime number factorization and it is a captivating technique to transfer the data progressively without disturbing the quality originality of the secured message. Thus, cryptography is concerned with the mathematical techniques which can propose secure communications in the occurrence of malicious hackers. Only the sender and receiver know the plain text by the simple cryptosystem. Cryptography is required to securely store the patient's data in cloud, and cryptography requires some mathematical calculations which affects the power consumption of the battery. Decryption to extract the original data is done at two levels, that is, Level-I and Level-II, because the encrypted data had been stored on cloud by applying the Proxy reencryption technique in earlier work, as mentioned in literature survey. In our work, Identity Based Encryption Technique is utilized for uploading data into the cloud. The identity of the user is extracted initially to generate the public key. The public key is generated by utilizing a phone number and e-mail ID. The obtained results depicted that the proposed approach offers better performance than the existing methods. The existing techniques with which the proposed scheme has been compared are GA07B [25], LZD [26], WCW [27], and IBPRE [28].

### 3.1. Role-Based Proxy Decryption Level-II Process

Using a decryption technique, encrypted data may be recovered from the original data, and decryption is used for security [29, 30]. The decryption contains the key to regaining access to the encrypted data. Data transmission handles the decryption procedure entirely. The data cannot be decrypted if the key does not match.

#### 3.1.1. Key Generation

Key creation is required for decryption. Cryptographic tools are employed during the process of establishing a key that incorporates the alpha-numeric sequence [31]. Key generation is the primary focus of software privacy. Unless the key is generated during encryption, no data can be decrypted because it is not generated during decryption. Before selecting any two attributes, data and user attributes are first extracted. In order to choose attributes, the AND operation is used. AND returns the value that is the local key. The private key is formed by performing an exclusive OR (XOR) operation on the local key [32]. Hashing is used to transform the private key into a secret key. Data retrieval requests from users are sent through third-party providers to the data owner. As soon as they had the secret key, users could decrypt whatever ciphertext [33] they had obtained from the cloud and get it back as their own personal plaintext.

#### 3.1.2. Key Authentication

Access to cryptographic key material should be given to the identified communication authority, and it is the communication entry between two parties. The two parties can be unilateral or mutual [34].

#### 3.1.3. Key Conformation

Communication agents should prove the control of authenticated keying material after providing key confirmation by the protocol [35].

#### 3.1.4. Key Freshness

Creating the unique and new independent generated keys among the different communication agents that grow the security and the process is done by key freshness. Generally, decryption algorithm robustness is considered according to the subsequent concerns, Compression friendliness, Security, Format compliance, and Time efficiency [36, 37].

Scalability in key management, privacy exposure risk, flexible access, and user revocation were some of the shortcomings of the technology used in electronic health records [38]. The protection of sensitive data is the primary concern when handling it. Because of this, a new algorithm is presented to explain it.

### 3.2. Work Flow

**Figure 1:**
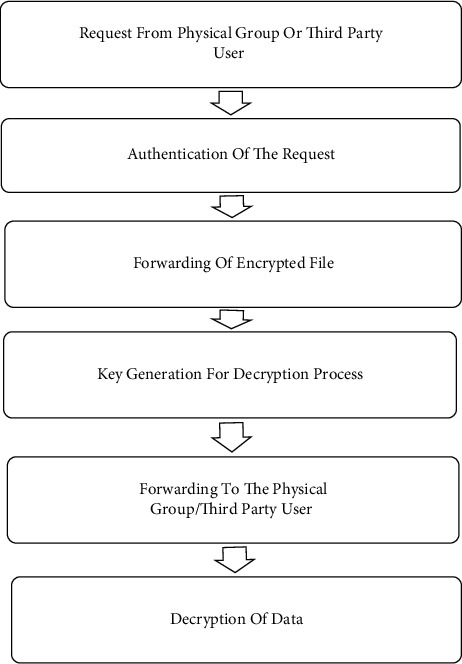
Flow of role-based proxy decryption level II.

The workflow of our proposed algorithm for role-based proxy decryption at level II is depicted in Figure 1. In the workflow, first of all, the third-party user will give a request to access the data that is the record of patients [39]. Request from the physical group or third party to access the user is given as the input for the decryption algorithm. Then the authentication process is carried out by checking the identity of the third party. After the successful authentication, encrypted file is forwarded to the user dependent.

In the role-specific model, the request received is from the nurse, lab technicians, or physician. A public key is generated as per the user's request and forwarded to the physician group. Third parties or physician group can access the data by utilizing the decryption key. Proxy decryption is introduced to extract the data, and again the decryption process is handled to extract the original data by using a public key so the medical data related to the patient can be securely retrieved by this mechanism.

### 3.3. Role-Based Proxy Decryption Level-II Proposed Algorithm

In our proposed methodology, an identity-based encryption technique is utilized for uploading data into the clouds. The identity of the user is extracted initially to generate the public key. The public key is generated by utilizing a phone number and e-mail ID. Finally, the encrypted data is uploaded to the clouds. The decryption of data is explained through a given novel proposed algorithm. Following are the symbols used in the algorithm for various parameters:

(i) The user (*U*_*i*_) holds four records,
  - Personal sensitive data is mentioned as *α*
  - Medical data is mentioned as *β*
  - Clinical reports are mentioned as *γ*
  - Detail of insurance policy is mentioned as *δ*
(vi) *P*_*G*_ is the physician group who are also registered with the cloud to get access to the users registered. There are three roles played in *P*_*G*_:
  - Physicians (*G*_*Py*_)
  - Nurse (*G*_*N*_)
  - Lab technicians (*G*_*Lb*_)
(vii) Third-party community service group (TP_*G*_) is the insurance personnel who provide funding to the patients if required.
(viii) Pr_*X*_ is the private key of the user, either the physician group user or the third party user.

## 4. Results and Discussion

The cloud storage for health care systems is used to implement our suggested EHR storage model, where each transaction is kept on a cloud and the energy usage for each transaction is computed [23]. In order to conduct the experiment, Java and Wamp server are used. The setup is done on an Intel(R) i3 CPU 2.50 GHz with 4 GB of RAM and 1 TB of local storage running Windows x64. In this work, simulation results of the existing and proposed algorithm are evaluated and compared on the basis of decryption time and energy consumed. Third parties, such as insurance agents, doctors, nurses, and others, can extract data relevant to them using an encryption key provided by the patient in the patient's health record system, which is used for encryption. This section provides the performance evaluation of our proposed approach in terms of level-2 decryption. The time of decryption could also be reduced in this proposed approach which makes the system a much more efficient one. If the user id and passwords are mismatched, then the user cannot open their page. This is required for the authentication of the user who is requesting data. The login window will be opened by registered doctors. It includes the user Id, password, and profession. Here the doctor enters all details and then uses the login button to open the pages. Similarly, other registered users like nurses or lab technicians can log in to access the data of patients. There will be an option to select the available patients and it includes different options like sending a request to Cloud Service Provider (CSP), getting a key, decrypting data, and extracting original data. Initially, the doctor sends the request to Cloud Service Provider (CSP). So, the doctors select the user and send a request to Cloud Service Provider (CSP). The cloud receives the request, and then it will show the details like request from id.dcr1, patient id.ptn1, and the profession of the person who has requested the data. The doctor sends the request to Cloud Service Provider (CSP) and gets the key from the sever.

### 4.1. Performance Analysis of Decryption Time Level-II

The performance analysis of decryption level-II time for different data sizes is discussed and presented.  Table 1 shows the performance analysis of checking decryption time for different data size of the proposed scheme with existing schemes.

**Figure 2:**
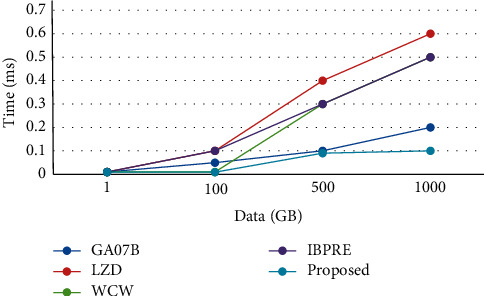
Performance analysis of decryption time Level-II.

Table 1 shows the performance analysis of decryption checking time level-I for different data sizes such as 1 GB, 100 GB, 500 GB, and 1000 GB. For validating and comparing simulation results to our suggested technique, four current methods are used: GA07B, LZD, WCW, and IBPRE. Aside from that, data with a size ranging from 1 GB to 1000 GB are employed to evaluate the performance of our proposed method. For 1 GB data size, the existing GA07B along with LZD, WCW, and IBPRE has taken time 0.01 ms to process the 1 GB data, whereas our proposed approach takes 0.009 ms only. For 100 GB data size, the existing GA07B takes much more time than the LZD, WCW, and IBPRE, whereas our proposed approach takes 0.009 ms only. For 500 GB data size, the existing LZD takes much more time than the three ones, whereas our proposed approach takes less time, 0.09 ms. Similarly, for 1000 GB data size, the existing LZD takes much more time than the GA07B WCW, IBPRE, whereas our proposed approach takes very less time, 0.01 ms only. From the obtained results, it is observed that the proposed method achieves better performance than the existing methods. The graphical representation of this performance evaluation of decryption level-II time for different data sizes is depicted in Figure 2.

### 4.2. Energy Consumption

**Figure 3:**
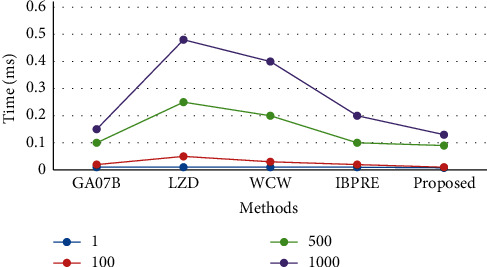
Performance analysis of consumed energy  ^*∗*^10 ^∧^5.

Table 2 shows the performance analysis of checking energy consumption with respect to time for different data sizes. Energy consumed is directly related to time. For validating and comparing the results, GA07B, LZD, WCW, and IBPRE are used. In addition, data of a size ranging from 1 to 1000 GB is used to evaluate the suggested approach's performance. The graphical representation of this evaluation is shown in Figure 3. The results depicted that the proposed approach offers better performance than the existing techniques. As it gives results in less time as compared to other methods and corresponding it will also consume less energy.

## 5. Conclusion

The main goal of this proposed work is to secure the intimate patient information by the deployment of the Electronic Health Record and to provide the better security in the EHRs using Encryption, Proxy based Reencryption, Level-I decryption, Level-II decryption. In this paper, cryptography methods are utilized for privacy, security, and reliability with E-health cloud systems through cloud storage systems. Several methods of E-health, decryption framework and personal health records are analyzed in a detailed way. Here, we proposed a scheme as a role-based proxy decryption approach that supported the delegate of decryption rights to user's revocation. This paper provides the performance evaluation of our proposed approach in level-II decryption by using role-based proxy decryption to extract the data and again, the decryption process is controlled to extract the original data with the help of a public key. The energy consumption in decryption for each transaction could be reduced in our proposed system, which makes the system a more efficient one. The ultimate aim of this paper is to secure the intimate patient information so that the medical data about the patient could be safely and securely retrieved using electronic health records. The study of various energy efficient cryptographic algorithms provides a large scope in the future of Mobile Cloud Computing. There are many ways to extend this work. In future work, the researcher plans to explore a strategy for developing more efficient attribute-based signcryption schemes for mobile cloud computing devices that fulfils both the elements of digital signature and public key encryption at the same time.

## Figures and Tables

**Algorithm 1:**
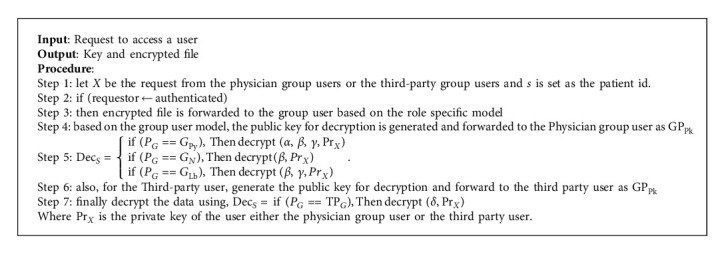
Role-based proxy decryption level-II.

**Table 1:** Performance analysis of decryption time Level-II  ^*∗*^10 ^∧^5.

Data (in GB)	GA07B	LZD	WCW	IBPRE	Proposed
1	0.01	0.01	0.01	0.01	0.005
100	0.01	0.1	0.01	0.1	0.005
500	0.2	0.1	0.2	0.2	0.1
1000	0.5	0.2	0.5	0.4	0.2

**Table 2:** Performance analysis of consumed energy  ^*∗*^10 ^∧^5.

Data in GB	GA07B	LZD	WCW	IBPRE	Proposed
1	0.01	0.01	0.01	0.01	0.008
100	0.02	0.05	0.03	0.02	0.01
500	0.1	0.25	0.2	0.1	0.09
*p*	0.15	0.48	0.4	0.2	0.13

## Data Availability

The data will be available from the authors upon request.

## References

[1] Bajpai V. (2014). The challenges confronting public hospitals in India, their origins, and possible solutions. *Advances in Public Health*.

[2] Fernando N., Loke S. W., Rahayu W. (2013). Mobile cloud computing: a Survey. *Future Generation Computer Systems*.

[3] Rahimi M. R., Ren J., Liu C. H., Vasilakos A. V., Venkatasubramanian N. (2014). Mobile cloud computing: a survey, state of art and future directions. *Mobile Networks and Applications*.

[4] Saab S. A., Chehab A., Kayssi A. Energy efficiency in mobile cloud computing: total offloading selectively works. Does selective offloading totally work?.

[5] Sanaei Z., Abolfazli S., Gani A., Buyya R. (2014). Heterogeneity in mobile cloud computing: taxonomy and open challenges. *IEEE Communications Surveys & Tutorials*.

[6] Rehaman M., Gao J., Tsai W. T. Energy savings in mobile cloud computing.

[7] Ruangchaijatupon N., Krishnamurthy P. Encryption and Power Consumption in Wireless LANs-N.

[8] Sivan R., Zukarnain Z. A. (2021). Security and privacy in cloud-based E-health system. *Symmetry*.

[9] Sharma P., Moparthi N. R., Namasudra S., Shanmuganathan V., Hsu C. H. (2021). Blockchain‐based IoT architecture to secure healthcare system using identity‐based encryption. *Expert Systems*.

[10] Tari Z., Yi X., Premarathne U. S., Bertok P., Khalil I. (2015). Security and privacy in cloud computing: vision, trends, and challenges. *IEEE Cloud Computing*.

[11] Jin L., Zhang Y., Chen X., Xiang Y. (2018). Secure attribute-based data sharing for resource-limited users in cloud computing. *Computers & Security*.

[12] Huang Q., Yue W., He Y., Yang Y. (2018). Secure identity-based data sharing and profile matching for mobile healthcare social networks in cloud computing. *IEEE Access*.

[13] Kalaiprasath R., Elankavi R., Udayakumar R. (2017). Cloud. Security and compliance- A semantic approach in end-to-end security. *International Journal on Smart Sensing and Intelligent Systems Special Issue*.

[14] Luna J., Taha A., Trapero R., Suri N. (2017). Quantitative reasoning about cloud security using service level agreements. *IEEE Transactions on Cloud Computing*.

[15] Genç Y., Afacan E. Identity-based encryption in the internet of Things.

[16] Zhang Y., He D., Obaidat M. S., Vijayakumar P., Hsiao K.-F. (2021). Efficient identity-based distributed decryption scheme for electronic personal health record sharing system. *IEEE Journal on Selected Areas in Communications*.

[17] Aljawarneh S. A., Yassein M. B. (2016). A conceptual security framework for cloud computing issues. *International Journal of Intelligent Information Technologies*.

[18] Shen Q., Liang X., Shen X., Lin X., Luo H. Y. (2014). Exploiting geo-distributed clouds for a e-health monitoring system with minimum service delay and privacy preservation. *IEEE journal of biomedical and health informatics*.

[19] Tseng Y., Fan C. I. (2021). Anonymous multireceiver identity-based encryption against chosen-ciphertext attacks with tight reduction in the standard model. *Security and Communication Networks*.

[20] Cao C., Tang Y., Huang D., Gan W., Zhang C. (2021). IIBE: an improved identity-based encryption algorithm for WSN security. *Security and Communication Networks*.

[21] Alornyo S., Zhao Y., Zhu G., Xiong H. (2020). Identity based key-insulated encryption with outsourced equality test. *International Journal on Network Security*.

[22] Deng H., Qin Z., Wu Q., Guan Z., Deng R. H., Wang Y. (2020). Identity-based encryption transformation for flexible sharing of encrypted data in public cloud. *IEEE Transactions on Information Forensics and Security*.

[23] Shao C., Yang Y., Juneja S., GSeetharam T. (2022). IoT data visualization for business intelligence in corporate finance. *Information Processing & Management*.

[24] Dhankhar A., Juneja S., Juneja A., Bali V. (2021). Kernel parameter tuning to tweak the performance of classifiers for identification of heart diseases. *International Journal of E-Health and Medical Communications*.

[25] Green M., Ateniese G. (2007). Identity based proxy Re-encryption. *Lecture Notes in Computer Science*.

[26] Lai J. Z., Zhu W. T., Deng H. (2010). New constructions for identity-based unidirectional proxy re-encryption. *Journal of Computer Science and Technology*.

[27] Weng J., Chen M., Yang Y., Deng R. H., Bao F. (2010). CCA-secure unidirectional proxy Re-encryption in the adaptive corruption model without ran-dom oracles. *Science China Information Sciences*.

[28] Wang X. A., Ma J., Zhang M., Xhafa F., Luo X. (2017). Cost-effective secure E-health cloud system using identity based cryptographic techniques. *Future Generation Computer Systems*.

[29] Liang K., Liu J. K., Wong D. S., Susilo . (2014). An efficient cloud-based revocable identity-based proxy Re-encryption scheme for public clouds data sharing. *European Symposium on Research in Computer Security*.

[30] Liu J. (2019). Identity Based Cryptography. http://www.securityboulevard.com.

[31] Li J., Zhang Y., Chene X., Xiangef Y. (2018). Secure attribute-based data sharing for resource-limited users in cloud computing. *Computers & Security*.

[32] Juneja A., Juneja S., Bali V., Mahajan S. (2021). Multi-criterion decision making for wireless communication technologies adoption in IoT. *International Journal of System Dynamics Applications*.

[33] Upadhyay H. K., Juneja S., Maggu S., Dhingra G., Juneja A. (2021). Multi-criteria analysis of social isolation barriers amid COVID-19 using fuzzy AHP. *World Journal of Engineering*.

[34] Rashid J., Kanwal S., Kim J., Nisar M. W., Naseem U. (2022). Heart disease diagnosis using the brute force algorithm and machine learning techniques. *Computers, Materials & Continua*.

[35] Kanwal S., Rashid J., Kim J. An attribute weight estimation using particle swarm optimization and machine learning approaches for customer churn prediction.

[36] Fatima M., Nisar M. W., Rashid J., Kim J., Kamran M., Hussain A. (2021). A novel fingerprinting technique for data storing and sharing through clouds. *Sensors*.

[37] Saleem K., Saleem M., Zeeshan R. (2022). Situation-aware BDI reasoning to detect early symptoms of covid 19 using smartwatch. *IEEE Sensors Journal*.

[38] Pandya S., Gadekallu T. R., Reddy P. K., Wang W., Alazab M. (2022). Infused Heart: a novel knowledge-infused learning framework for diagnosis of cardiovascular events. *IEEE Transactions on Computational Social Systems*.

[39] Arikumar K. S., Prathiba S. B., Alazab M. (2022). FL-PMI: federated learning-based person movement identification through wearable devices in smart healthcare systems. *Sensors*.

